# Mutations in AraR leading to constitutive of arabinolytic genes in *Aspergillus niger* under derepressing conditions

**DOI:** 10.1007/s00253-019-09777-0

**Published:** 2019-04-08

**Authors:** Jos Reijngoud, Malte Deseke, Elmar T. M. Halbesma, Ebru Alazi, Mark Arentshorst, Peter J. Punt, Arthur F. J. Ram

**Affiliations:** 1 Present Address: Molecular Microbiology and Biotechnology, Institute of Biology Leiden, Leiden University, Sylviusweg 72, 2333 BE Leiden, The Netherlands; 2 Dutch DNA Biotech, Hugo R Kruytgebouw 4-Noord, Padualaan 8, 3584 CH Utrecht, The Netherlands

**Keywords:** Transcriptional regulation, Plant cell wall, Arabinases, Carbon catabolite repression, creA

## Abstract

The AraR transcription factor of *Aspergillus niger* encodes a Zn(II)_2_Cys_6_ transcription factor required for the induction of genes encoding arabinolytic enzymes. One of the target genes of AraR is *abfA*, encoding an arabinofuranosidase. The expression of *abfA* as well as other L-arabinose-induced genes in *A. niger* requires the presence of L-arabinose or its derivative L-arabitol as an inducer to activate AraR-dependant gene expression. In this study, mutants were isolated that express L-arabinose-induced genes independently of the presence of an inducer under derepressing conditions. To obtain these mutants, a reporter strain was constructed in a *ΔcreA* background containing the L-arabinose-responsive promoter (*PabfA*) fused to the acetamidase (*amdS*) gene. Spores of the *ΔcreA PabfA-amdS* reporter strain were UV-mutagenized and mutants were obtained by their ability to grow on acetamide without the presence of inducer. From a total of 164 mutants, 15 mutants were identified to contain transacting mutations resulting in high arabinofuranosidase activity in the medium after growth under non-inducing conditions. Sequencing of the *araR* gene of the 15 constitutive mutants revealed that 14 mutants carried a mutation in AraR. Some mutations were found more than once and in total nine different point mutations were identified in AraR. The AraR^N806I^ point mutation was reintroduced into a parental strain and confirmed that this point mutation leads to inducer-independent expression of AraR target genes. The inducer independent of L-arabinose-induced genes in the AraR^N806I^ mutant was found to be sensitive to carbon catabolite repression, indicating that the CreA-mediated carbon catabolite repression is dominant over the AraR^N806I^ mutant allele. These mutations in AraR provide new opportunities to improve arabinase production in industrial fungal strains.

**Electronic supplementary material:**

The online version of this article (10.1007/s00253-019-09777-0) contains supplementary material, which is available to authorized users.

## Introduction

*Aspergillus niger* is a saprobic fungus which produces a large arsenal of plant cell polymer-degrading enzymes. Most noticeably, these include enzymes involved in degradation of plant storage polymers such as starch and inulin, as well as plant cell wall polymers such as cellulose, hemicellulose, and pectin (Pel et al. 2007; de Vries et al. 2017). D-Xylose and L-arabinose are the most abundant pentoses in the plant cell wall. L-Arabinose is found in both hemicellulose (as arabinoxylan) and pectin (as arabinogalactan and arabinan) (McNeil et al. 1984). Filamentous fungi, including *A. niger*, produce a number of extracellular arabinases, including α-L-arabinofuranosidases (ABFs) and endo-arabinases (ABNs), to degrade L-arabinose-containing plant cell wall polymers (de Vries and Visser 2001). Following uptake of L-arabinose, its intracellular catabolism involves the pentose catabolic pathway which consists of a five-step metabolic route resulting in the formation of D-xylulose 5-phosphate, which is further metabolised in the pentose phosphate pathway (Khosravi et al. 2015). Several ABFs, ABNs, and L-arabinose-specific catabolic pathway genes, including the L-arabinose reductase (LarA), L-arabitol dehydrogenase (LadA), and L-xylulose reductase (LxrA), are coordinately expressed (Flipphi et al. 1994; de Groot et al. 2003; Battaglia et al. 2014). The last two catabolic steps in L-arabinose catabolism connect with D-xylose catabolism and include xylitol dehydrogenase (XdhA) and D-xylulose kinase (XkiA) to form D-xylulose 5-phosphate (de Groot et al. 2007). Efficient extracellular degradation of L-arabinose-containing polymeric substrates and subsequent catabolism requires a coordinated expression of the genes encoding these enzymes.

In *A. niger*, the coordinated expression of genes encoding the extracellular enzymes (ABFs and ABNs) and intracellular enzymes (LarA, LadA, LxrA, XdhA, and XkiA) in response to the presence of L-arabinose requires the Zn(II)_2_Cys_6_ transcription factor AraR (Battaglia et al. 2011, 2014). The L-arabinose-responsive transcription factor AraR shows a strong sequence similarity to the D-xylose-responsive Zn(II)_2_Cys_6_ transcription factor XlnR and evolutionary analysis suggests that AraR is derived from XlnR after gene duplication (Battaglia et al. 2011). Analysis of the growth phenotypes of single knockout mutants of *araR* and *xlnR* and the double knockout mutant on different substrates suggests partially overlapping roles for AraR and XlnR (Battaglia et al. 2011). Expression of the last two genes in the pentose catabolic pathway (*xdhA* and *xkiA*) as well as genes in the pentose phosphate pathway are not only induced on L-arabinose via AraR, but also induced on D-xylose via the D-xylose-responsive transcription factor XlnR (Battaglia et al. 2011). The two positive regulators of the pentose metabolism (XlnR and AraR) both have specific target genes, but some genes can also be under the control of both transcription factors (Kowalczyk et al. 2015; Gruben et al. 2017). Detailed comparison of the involvement of AraR and XlnR in the induction of pentose catabolic genes in *A. oryzae* has recently shown that all pentose catabolic pathway genes are under the control of both AraR and XlnR, except *larA* which is under the control of only AraR (Ishikawa et al. 2018). DNA-binding studies revealed that XlnR and AraR bind to similar, but not identical DNA sequences. XlnR prefers CGGNTAAW, while AraR prefers CGGDTAAW (Ishikawa et al. 2018).

The AraR transcription factor of *A. niger* is an 832 amino acid long protein and conserved domains include the binuclear cluster DNA-binding domain (Zn(II)_2_Cys_6_) (aa 32–73) and a domain indicated as the fungal-specific transcription factor domain (also known as middle homology region) (aa 496–736) (Battaglia et al. 2011). While the DNA-binding function of the Zn(II)_2_Cys_6_ domain is well established, the function of the conserved middle homology region is not clear. The mechanism by which AraR is activated in response to the presence of an inducer is also unknown. Since the accumulation of intracellular L-arabitol results in higher production of arabinolytic enzymes, L-arabitol is regarded as the inducer to activate AraR (van der Veen et al. 1993). L-Arabinose- or L-arabitol-induced gene expression and production of extracellular enzymes (arabinofuranosidases (AbfA, AbfB), endo-arabinase (AbnA)) and intracellular enzymes (L-arabinose reductase, L-arabitol dehydrogenase, L-xylulose reductase, and xylitol dehydrogenase) have been shown to be strongly repressed by D-glucose in a CreA-dependent manner (van der Veen et al. 1994; Flipphi et al. 1994).

The filamentous fungus *A. niger* is the main producer of arabinan-degrading enzymes in industry. The enzymes are used in several industrial applications including improvement of wine flavours, juice clarification, pulp treatment, and plant biomass degradation (Numan and Bhosle 2006; Shallom and Shoham 2003). The production of arabinan-degrading enzymes is tightly controlled and requires the presence of inducing sugars or polyols like L-arabinose or L-arabitol and the absence of repressing sugars like D-glucose. The requirement of specific inducers and the sensitivity to carbon catabolite repression limits the choice of feedstock and the use of cheap feedstocks for arabinase production.

In this study, we have isolated mutants with constitutive production of arabinases that do not require the presence of L-arabinose or L-arabitol as inducers. Mutants were found to have specific amino acid mutations in the AraR transcriptional activator resulting in a constitutively active form of the transcription factor, bypassing the inducer requirement. Detailed analysis of one of the AraR constitutive mutants (AraR^N806I^) revealed that the constitutive production of arabinases is still sensitive to carbon catabolite repression.

## Material and methods

### Strains and growth conditions

The *A. niger* strains used in this study are listed in Table 1. *S*trains were grown on either solidified (2% agar) or liquid minimal medium (MM) or complete medium (CM). MM contains 7 mM KCl, 8 mM KH_2_PO_4_, 70 mM NaNO_3_, 2 mM MgSO_4_, and spore trace element solution, pH 5.5 (Arentshorst et al. 2012). The carbon source of interest was added at a final concentration of 1%. If L-arabinose was the carbon source, 0.05% of D-glucose was added to promote germination of the spores. For CM, 0.1% casamino acids and 0.5% yeast extract were added to MM and 1% D-glucose was added as a carbon source. MM-agar medium containing 10 mM acetamide as the sole nitrogen source and 50 mM D-glucose as carbon source were made as described previously (Arentshorst et al. 2012). For spore propagations, spores were streaked on CM-agar plates with a cotton stick and incubated at 30 °C for 3 to 5 days. The spores were isolated by adding 10 to 15 ml of 0.9% NaCl solution and scrubbed with a cotton stick. This spore suspension was taken out of the Petri dish, filtered through sterile miracloth tissue, and the number of spores was counted with a TC20™ automated cell counter (Bio-Rad, Veenendaal, the Netherlands). The spores were stored in 0.9% NaCl at 4 °C. Five litres of bioreactor cultivation (New Brunswick Scientific, Nijmegen, the Netherlands) was performed as previously described using 0.75% D-fructose as carbon source (Niu et al. 2017).

**Table 1:** Strains used in this study

Strain	Genotype	Reference
N402	*cspA*	ATCC® 64974™; Bos et al. 1988
AB4.1	*pyrG* derivative of N402	Van Hartingsveldt et al. 1987
JN11.2	PabfA-amdS-pyrG* in AB4.1	Niu et al. 2015
JN16.1	*creA::hygB* in JN11.2	Niu et al. 2015
MA323.1	*ku70::amdS ∆nicB ∆pyrG*	Niu et al. 2016
JR12.3	*araR*^*wt*^-pyrG** in AB4.1	This study
JR13.9	*araR*^*N806I*^-pyrG** in AB4.1	This study
JR13.2	*araR*^*N806I*^-pyrG** in AB4.1 + multiple copies of *araR*^*N806I*^ randomly integrated	This study
JN16.1 U#7	UV mutated JN16.1	This study
EH1.2	*araR::nicB* in MA323.1	This study
EH2.1	*araR*^*WT*^-pyrG** in EH1.2	This study
EH3.1	*araR*^*N806I*^-pyrG** in EH1.2	This study
JR16.2	*creA::hygB* in EH2.1	This study
JR17.2	*creA::hygB* in EH3.1	This study

To analyse the growth phenotypes of the strains, 5 μl of spore suspension at a concentration of 1 × 10^5^ spores/ml was point-inoculated in the middle of an agar plate containing MM and the nitrogen and carbon sources to be assayed. The plates were incubated at 30 °C for 4 days. For shaken liquid cultures, flasks with 50 ml MM containing the carbon source of interest were inoculated with the spore stock solution to reach a final spore concentration of 1 × 10^6^ spores/ml. The flasks were incubated at 30 °C, 250 rpm for 36 h in the Innova®44 incubator (New Brunswick Scientific, Nijmegen, the Netherlands). The mycelia were separated from the growth medium by filtration and immediately frozen in liquid nitrogen. The growth medium, referred to as supernatant of the culture, was collected in a 15-ml tube, 5 × 1 ml aliquots were taken, and both tube and aliquots were immediately frozen in liquid nitrogen as well. These samples were stored together with the mycelia at − 80 °C for long-term storage.

The UV-mutagenesis experiments were performed as described previously (Damveld et al. 2008) using JN16.1 (*PafbA-amdS ΔcreA*) (Niu et al. 2015) as a starting strain. Surviving spores (70%) were plated out on MM-agar containing acetamide as sole nitrogen source and 50 mM D-glucose as a carbon source. On each plate, about 2.5 × 10^5^ spores (50 μl of a 5 × 10^6^ spores/ml) were plated out. In total, 40 plates were prepared and incubated at 30 °C for 7 days. As the survival rate was about 70%, about 7 × 10^6^ spores were screened for growth on acetamide plates indicating constitutive expression of *abfA.* In total, 160 mutants were obtained. In addition, spontaneous mutants were selected by plating out non-UV-treated spores at MM-acetamide/D-glucose medium. On four plates, this yielded to four mutants. All mutants were purified twice on MM-agar plates with acetamide/D-glucose and resulted in the isolation of in total 164 mutants.

### Plasmid and strain construction

Transformations were carried out as described previously (Arentshorst et al. 2012). AraR was knocked out using the split marker approach (Arentshorst et al. 2015a) in which the open reading frame of *araR* is replaced by the *nicB* gene of *A. nidulans* in MA323.1 (*∆ku70*, *nicB*^*−*^, *pyrG*^*−*^). The *nicB* gene encodes a nicotinate mononucleotide pyrophosphorylase and can be used as an auxotrophic marker as the *nicB* mutant can be supplemented with nicotinamide to grow (Niu et al. 2016). Primers for constructing gene deletion fragments and diagnostic PCRs are listed in Supplemental Table S1. Proper deletion of *araR* in the resulting strain (EH1.2) was confirmed by diagnostic PCR and Southern blot (Supplemental Fig. S1). Subsequently, EH1.2 and AB4.1 were used to integrate an *araR* allele (wild type or N806I) at the *pyrG* locus.

The *araR*^*WT*^ and *araR*^*N806I*^ alleles including the 1-kb flanks were PCR amplified using primers with a *Not*I site and cloned in pJET (Supplemental Table S1). The *araR* fragments were isolated from the vectors with *Not*I and ligated in *Not*I-digested pyrG** targeting vector pMA334 (Arentshorst et al. 2015b) resulting in pMA334-araR^WT^ and pMA334-araR^806^. After verification by restriction analysis and sequencing, pMA334-araR^WT^ and pMA334-araR^806^ were *Asc*I digested to obtain linear fragments for transformation of AB4.1 and EH1.2 using the auxotrophic *pyrG* marker as selection marker. Proper integration of pMA334-araR^WT^ and pMA334-araR^806^ at the *pyrG* locus in EH1.2 was confirmed by Southern blot analysis (Supplemental Fig. S2). The *creA* gene was deleted in EH2.1 (*ΔaraR araR*^*WT*^) and EH3.1 (*ΔaraR araR*^*N806I*^) using split marker fragments with hygromycin resistance gene as a selection marker using primers listed in Supplemental Table S1. The diagnostic PCRs to confirm deletion of *creA* are given in Supplemental Fig. S3. Proper integration of pMA334-araR^WT^ and pMA334-araR^806^ at the *pyrG* locus in AB4.1 was confirmed by Southern blot analysis (Supplemental Fig. S4).

### Enzyme activity assays

Arabinofuranosidase activity in the supernatant of the liquid cultures was detected with 4-nitrophenyl α-L-arabinofuranoside (Sigma-Aldrich, Steinheim, Germany) essentially as described by Tefsen et al. (2012).

For the screening of the 164 mutants, spores were picked from a plate with a sterile toothpick and transferred to a sterile 96-well plate filled with 200 μl of MM with 1% D-glucose. Plates were incubated for 36 h at 30 °C. From each microtitre well, 2.5 and 7.5 μl of cell-free growth medium were transferred to a fresh microtitre plate and measured for arabinofuranosidase activity. Arabinofuranosidase activity was measured by mixing supernatant with 44 μl 0.1 M sodium acetate buffer (pH 4.5) and 6 μl of 5 mM 4-nitrophenyl α-L-arabinofuranoside in a flat-bottom well of a 96-well plate (Sarstedt, Etten-Leur, the Netherlands). The final volume in each well was adjusted to 60 μl with water. The plate was incubated for 30 min at 37 °C and 240 μl 0.25 M sodium hydroxide was added to each well to stop the reaction. The absorbance was measured at a wavelength of 405 nm with the EnSpire® Multimode Plate Reader (Perkin Elmer, Rotterdam, the Netherlands). Selected strains showing arabinofuranosidase activity in the microtitre plate were also grown in shake flask cultures in 50 ml MM containing 1% D-glucose and incubated for 36 h at 30 °C at 250 rpm. Again, cell-free medium (2.5, 5.0, 7.5, or 10.0 μl) was measured for arabinofuranosidase activity as described above. Control strains (N402; wild type) or JN11.2 (*PabfB-amdS*) were also grown in L-arabinose medium to measure arabinofuranosidase activity under inducing conditions.

### General molecular procedures

The PCR amplification of the *araR* gene and its flanking regions for the sequencing experiments and that of the probes for the Southern and Northern blots was performed by using the Phire Hot Start II polymerase (Thermo Scientific, Breda, the Netherlands) according to instructions of the manufacturer using the Prime Thermal Cycler (Techne, (VWR) Amsterdam, the Netherlands) PCR machine. DNA isolation from the fungal mycelia frozen in liquid nitrogen was done according to the procedure described by Arentshorst et al. (2012) via a phenol-chloroform-isoamyl alcohol extraction. DNA extraction from agarose gels was performed using the GeneJET™ gel extraction kit from Fermentas Life Sciences (Amsterdam, the Netherlands) according to the manufacturer’s instructions. DNA concentrations were measured spectrophotometrically with the Thermo Scientific Nano Drop 2000 Spectrophotometer (Thermo Scientific, Breda, the Netherlands) at a wavelength of 260 nm. RNA extraction and Northern blot analysis were performed as described earlier (Alazi et al. 2016). Primers for the amplification of the probes are listed in Supplemental Table S1.

### Sequencing of *araR* and sequence analysis

Genomic DNA of selected constitutive mutants and the parental strain was isolated and the *araR* open reading frame and 1-kb flanking regions weres PCR amplified using primers AraRP1 and AraRP2 (Supplemental Table S1). The PCR product (4.5 kb) was purified from gel and sequenced at Macrogen Europe, Amsterdam, Netherlands. Sequence analysis was performed with the software programs Serial Cloner 2.6.1 (http://serialbasics.free.fr/Serial_Cloner.html) and DNAMAN Version 4.0 (https://lynnon.com/). The alignment of AraR and XlnR comparison was done using tools available at UniProt (www.uniprot.org).

## Results

### In vivo reporter strains to analyse induction and carbon catabolite repression mechanisms of the L-arabinose-induced arabinofuranosidase A

**Fig. 1:**
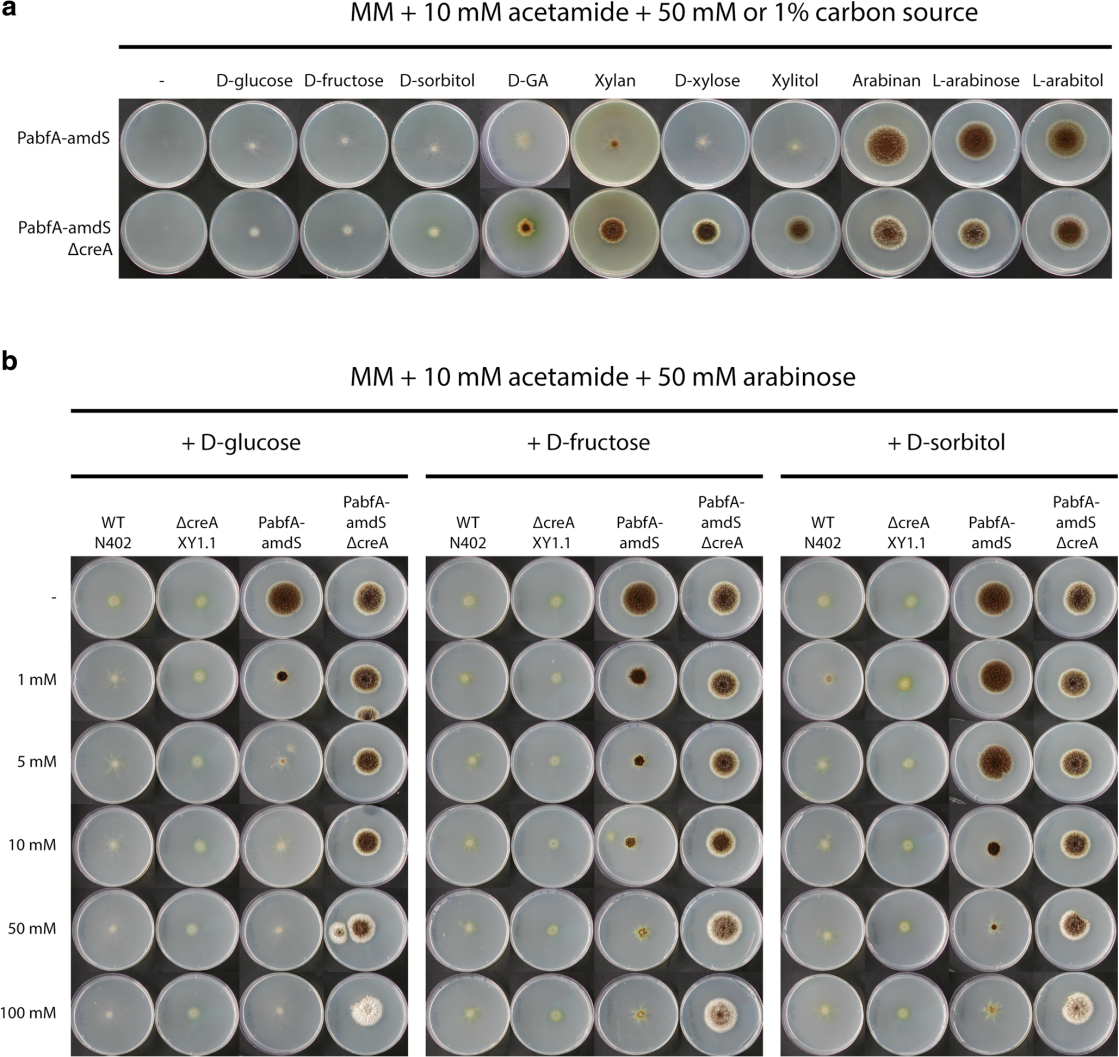
Growth analysis of *A. niger PabfA-amdS* reporter strains. **a** Induction of the *PabfA*-*amdS* reporter strains in a wild-type *creA* background (JN11.2) or in a *ΔcreA* background (JN16.1) is analysed by growing on MM with 10 mM acetamide with 50 mM monosaccharide or 1% polysaccharide. **b** Repression of the reporter strains is analysed by growing on MM with 10 mM acetamide with 50 mM L-arabinose as inducer and different concentrations of the repressing carbon sources, such as D-glucose, D-fructose, and D-sorbitol. A wild-type strain (N402) and a *creA* deletion strain (XY1.1) which do not contain the *Aspergillus nidulans amdS* gene are used as negative controls. Strains were grown for 7 days at 30 °C

We previously constructed a *PabfA-amdS* reporter strain (JN11.2) and showed that expression of *amdS* from the arabinofuranosidase A (*abfA*) promoter is specifically induced by L-arabinose allowing the reporter strain to grow on L-arabinose and acetamide as the carbon and nitrogen source, respectively (Niu et al. 2015). As shown in Fig. 1a, L-arabitol and arabinan also induced *abfA* expression. Other carbon sources such as D-glucose, D-fructose, D-sorbitol, D-galacturonic acid, and D-xylose did not result in growth of the reporter strain on acetamide, indicating that these carbon sources do not induce expression of the *amdS* gene from the *abfA* promoter (Niu et al. 2015; Fig. 1a, upper row).

In this study, we also tested whether disruption of the carbon catabolite repressor (*creA*) in the JN11.2 background affected the expression of *abfA* and consequently the growth on acetamide. As shown in Fig. 1a, lower row, strain JN16.1 (*ΔcreA::hygB PabfA-amdS*) grew on L-arabinose, L-arabitol, and arabinan, but also on D-xylose, xylitol, and xylan, and to a lesser extent on D-galacturonic acid (GA), indicating that D-xylose and possibly GA induce *abfA* expression under derepressing conditions. The *PabfA-amdS* reporter strain in the *ΔcreA* background did not grow on D-glucose, D-fructose, or D-sorbitol indicating that derepression via deletion of *creA* is not sufficient to drive *amdS* expression from the *abfA* promoter to sustain growth. As expected and as shown earlier (Niu et al. 2015), both the wild-type and *ΔcreA* mutants without the reporter constructs do not grow on acetamide plates on any carbon source (Fig. 1b).

Arabinofuranosidases, including *abfA*, have been shown to be under carbon catabolite repression control (van der Veen et al. 1993; de Ruijter et al. 1997). To examine the effect of carbon catabolite repression, spores of the *PabfA-amdS* reporter strain were inoculated on MM-acetamide plates containing L-arabinose as an inducer and increasing concentrations of D-glucose, D-fructose, or D-sorbitol (Fig. 1b). Monitored by the ability to grow on acetamide, the results indicate that the expression of *abfA* is strongly repressed by glucose at concentrations of 1 mM and higher. As shown in Fig. 1b, d, fructose and D-sorbitol addition also led to significant repression although the concentrations to mediate repression via D-fructose (10 mM) or D-sorbitol (50 mM) on plates are higher compared to glucose. Carbon repression was lost when the *creA* was deleted in the reporter strain (Fig. 1b).

### Isolation and sequence analysis of trans-acting mutants with constitutive expression of L-arabinose-induced genes

To select for mutants with constitutive expression of *abfA*, JN16.1 (*PabfA-amdS ΔcreA::hygB*) was UV-mutagenized and surviving spores (70% survival rate) were plated out on MM-agar plates with acetamide as the sole nitrogen source and D-glucose as a carbon source. In addition, four plates with non-UV-treated spores were plated to select for spontaneous mutants. In total, 160 UV mutants and four spontaneous mutants were isolated after two rounds of purification on MM-acetamide/D-glucose plates.

To identify trans-acting mutants, 20 mutants were tested for arabinofuranosidase activity by growing them in shake flask cultures with MM-D-glucose medium and analysing the supernatant for arabinofuranosidase activity. Of these 20 mutants, four displayed higher arabinofuranosidase activity compared to the parental strain. Next, the remaining 144 mutants were grown in MM-glucose medium in microtitre plates to assay total arabinofuranosidase activity. From the 144 mutants, 13 of them showed an arabinofuranosidase activity at least twofold above background resulting in a total of 17 mutants producing arabinofuranosidases under non-inducing conditions (data not shown). All mutants with higher arabinofuranosidase activity were obtained after UV mutagenesis.

**Fig. 2:**
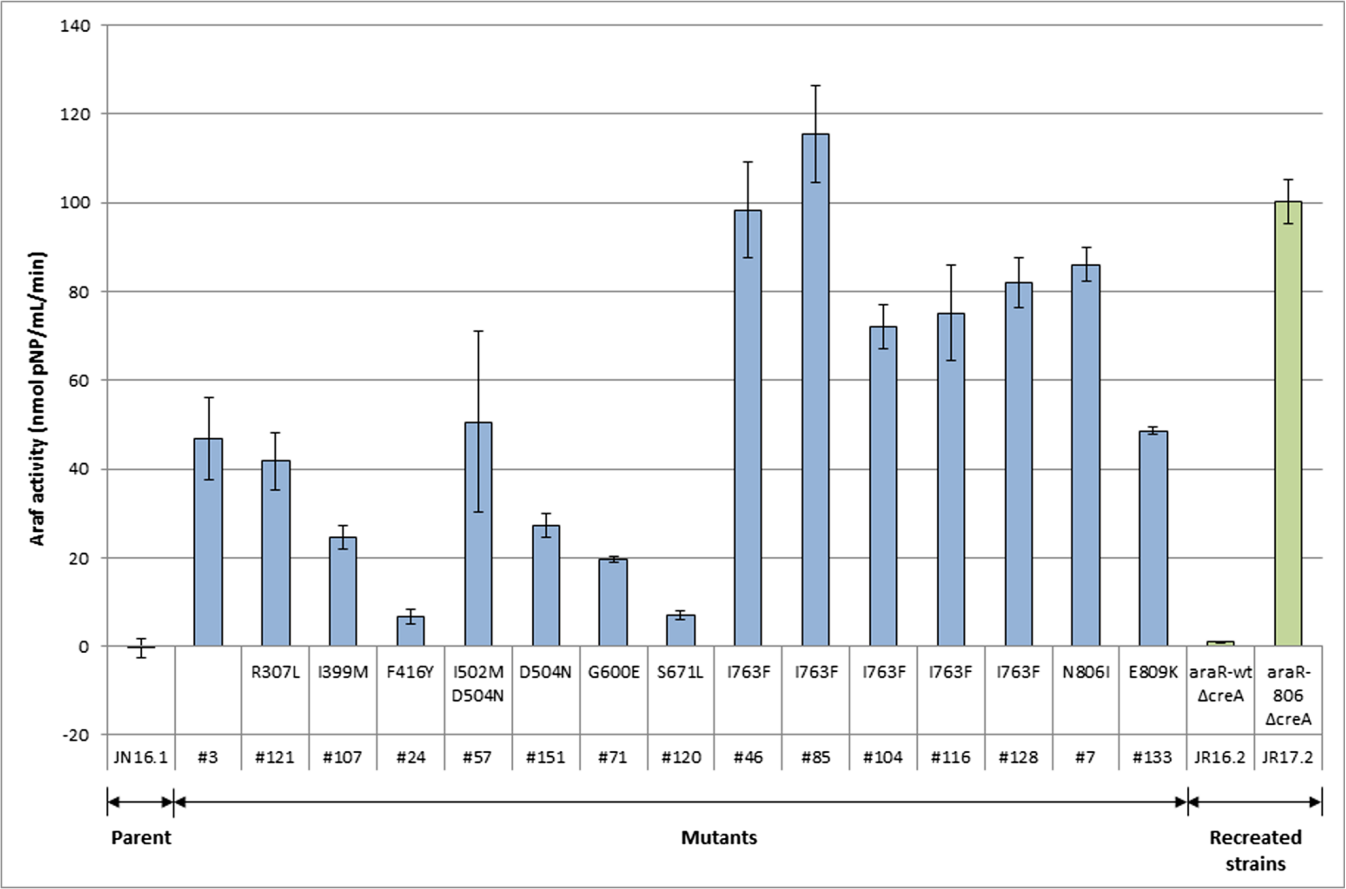
Arabinofuranosidase activity of the *A. niger* parental strain containing the *PabfA-amdS* reporter construct (JN16.1), the mutants derived from JN16.1 after UV mutagenesis (bars in blue), and the reconstructed *∆creA araR*^*WT*^ (JR16.2) and the reconstructed *∆creA araR*^*N806I*^ (JR17.2) strains (bars in green). All trains were grown in 50 ml MM-D-glucose medium supplemented with 0.0003% yeast extract for 36 h 30 °C at 250 rpm. Error bars represent the variation between biological duplicates

To determine whether mutations in *araR* were responsible for the constitutive expression of arabinolytic genes, the *araR* locus of the 17 mutants was PCR amplified and sequenced. In 16 out the 17 mutants, mutations in *araR* were found, indicating that the AraR transcription factor itself is the major target to obtain mutants constitutively expressing arabinases (Fig. 2). Two mutations (AraR^I763F^ and AraR^D504N^) were found in five and two mutants, respectively. In addition to these two mutations, seven additional mutations in *araR* were identified, all leading to an amino acid change in the protein sequence. Mutant #57 contained two mutations (I502M and D504N). Since constitutive mutant #151 contained only the D504N mutation, we cannot conclude whether the I502M mutation by itself also leads to constitutive expression, and therefore, the I502M mutation was not considered to be leading to constitutive phenotype. In mutant #3, no mutations were found in the *araR* gene, nor in the *xlnR* gene, and no mutations were found in the 1-kb upstream regions of both *araR* and *xlnR*. To compare arabinofuranosidase activities of the parental strain (JN16.1) and 17 constitutive mutants, strains were grown in duplicate under comparable conditions in shake flask cultures in MM-glucose for 36 h and the arabinofuranosidase activity was determined. Comparison of the arabinofuranosidase activities detected in the growth medium among the mutants identified the AraR^I763F^ (mutants #46, #85, #104, #116, and #128) and the AraR^N806I^ (mutant #7) as the AraR variants with the strongest constitutive expression (Fig. 2).

### The AraR^N806I^ mutation is responsible for the constitutive production of arabinofuranosidases

Because of its high arabinofuranosidase activity under non-inducing conditions, mutant #7 carrying the AraR^N806I^ mutation was selected for further analysis. To confirm that besides AraR^N806I^ no additional UV-introduced mutations were responsible for the constitutive phenotype, *araR* was knocked out (EH1.2) and subsequently complemented by *araR*^*WT*^ (EH2.1) or the constitutive *araR*^*N806I*^ allele (EH3.1) at the *pyrG* locus by targeted integration. Proper deletion of *araR* in EH1.2 was confirmed by phenotypic growth analysis on MM-L-arabinose agar plates (data not shown) and Southern blot analysis (Supplemental Fig. S1) and integration of either the wild-type *araR* allele or *araR*^*N806I*^ allele at the *pyrG* locus was confirmed by Southern blot (Supplemental Fig. S3). Subsequently, the *creA* gene was deleted in both strains resulting in strains JR16.2 and JR17.2 respectively (Supplemental Fig. S4). The reconstituted mutant strain JR17.2 showed constitutive arabinofuranosidase activity under non-inducing condition comparable to the original mutant, indicating that the AraR^N806I^ mutation is solely responsible for the constitutive phenotype (Fig. 2). Strain JR16.2 did not show arabinofuranosidase activity under non-inducing conditions as expected.

### The AraR^N806I^ mutation behaves as a semi-dominant mutation and is overruled by CreA-mediated repression during exponential growth

To determine whether the AraR^N806I^ mutation is dominant over the wild-type AraR, strain JR13.9 was constructed which, in addition to the *araR* wild-type gene at the endogenous locus, contains the AraR^N806I^ allele, expressed from its own promoter at the *pyrG* locus. As a control strain, JR12.3 was constructed which contains an extra copy of the wild-type *araR* gene at the *pyrG* locus. Correct and single-copy integration of *araR*^*WT*^ and *araR*^*N806I*^ was confirmed by Southern blot analysis and sequencing (Supplemental Fig. S2). In the Southern blot analysis of transformants, also a multicopy (mc) transformant (JR13.2) containing at least two copies of the AraR^N806I^ allele (mc-*araR*^*N806*I^) was identified and included in the analysis. As a control, we used strain EH3.1, which only contains the *araR*^*N806I*^ allele at the *pyrG* locus and is lacking the endogenous *araR* gene.

Because these four strains contain the wild-type *creA* gene, they were cultivated using D-fructose as a non-inducing, less-repressing carbon source. Maximum biomass accumulation and growth rate of the four strains were similar indicating that there were no growth differences between the strains (Table 2). Time points representing exponential phase (70% and 90% of maximal biomass accumulation) and time point representing stationary phase (20 h and 70 h after D-fructose depletion) were selected to determine arabinofuranosidase activity as described in the “Material and methods” section. No arabinofuranosidase activity was detected in any of the four strains during exponential growth, indicating constitutive expression is prevented during exponential growth possibly by carbon catabolite repressing mechanism even on D-fructose. Increased arabinofuranosidase activity was detected in strains carrying the mutant AraR^N806I^ allele during stationary phase (Table 2). The highest activity during stationary phase was found for the strain carrying multiple copies of AraR^N806I^ allele (JR13.2) or in the strain containing only the AraR^N806I^ allele (EH3.1). The strain containing both the wild-type *araR* gene and the mutant form (JR13.9) showed intermediate arabinofuranosidase activity 20 h after carbon depletion. The results suggest that the AraR^N806I^ allele is semi-dominant over the wild-type AraR allele.

To show that CreA-mediated repression is responsible for the repression of arabinofuranosidase activity during exponential growth in the strains expressing the AraR^N806I^ allele, the original AraR^N806I^ mutant (JN16.1#7, *araR*^*N806I*^*ΔcreA*) was cultured in bioreactor on D-fructose. The growth rate of this mutant, as well as the parental strain (JN16.1), was lower as compared to the wild-type due to *creA* deletion (Table 2). Arabinofuranosidase activity in JN16.1#7 was already detected during exponential phase, indicating that CreA represses arabinofuranosidase production during exponential growth in the strains carrying the AraR^N806I^ mutant allele even when grown on D-fructose.

**Fig. 3:**
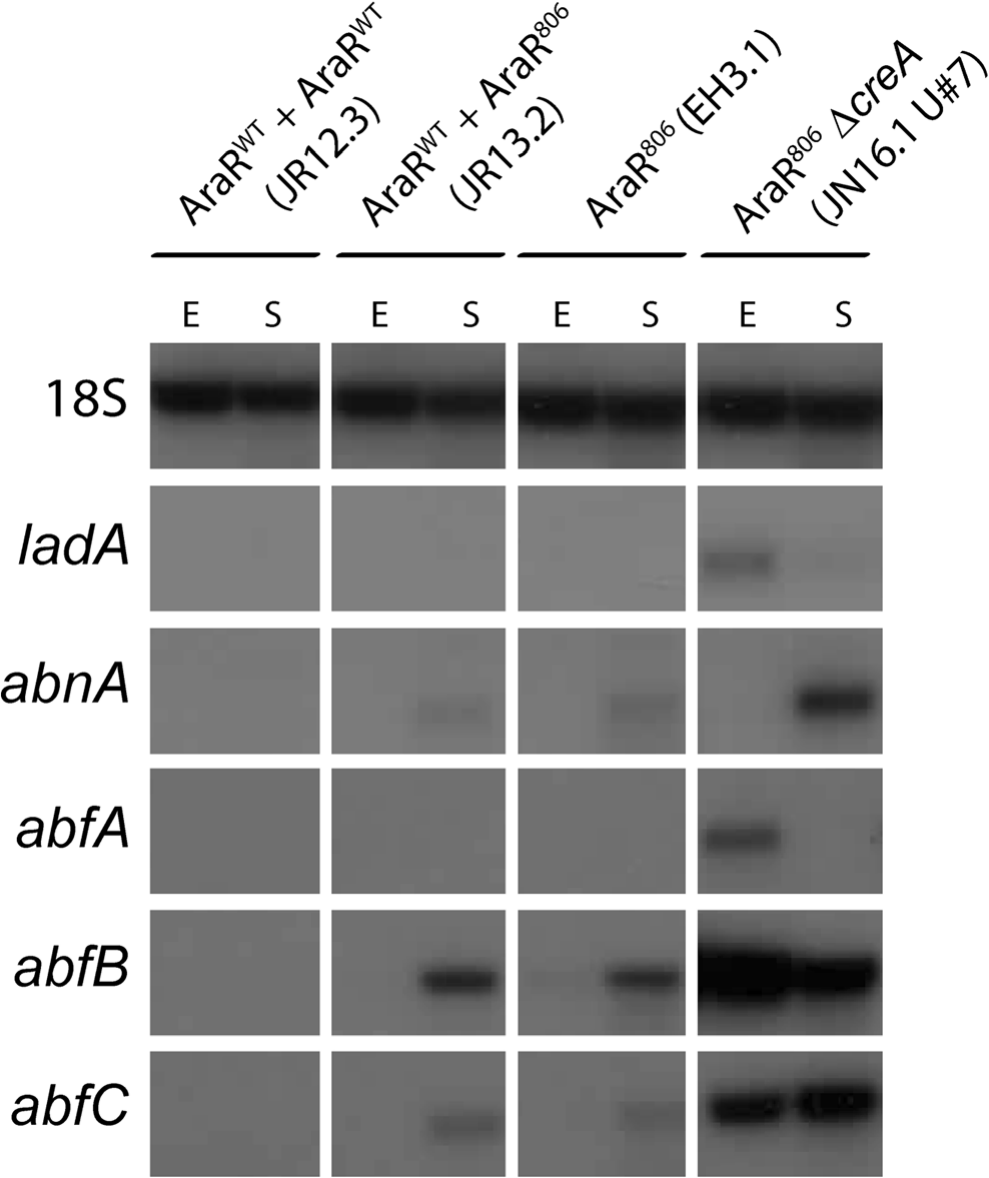
Expression analysis of L-arabinose-responsive genes in submerged batch cultivation at the end of exponential phase (E) and at stationary phase (S). Strains were grown in pH 3.0 controlled 5-l bioreactors containing minimal medium with 0.75% D-fructose as carbon source as described in the “Material and methods.” Throughout cultivation, mycelium samples were taken from the cultures and stored at − 80 °C. Samples corresponding with a biomass yield of 90% of the maximum biomass reached, or samples corresponding with the time point 20 h after fructose depletion, representing exponential (E) or stationary (S) phases respectively were used for RNA extraction and subsequent Northern blot analysis. Probes used include *ladA* (L-arabitol dehydrogenase; An01g10920), *abnA* (endo-1,5-alpha-arabinanase; An09g01190), and the arabinofuranosidases encoded by *abfA* (An01g00330), *abfB* (An15g02300), and *abfC* (An08g01710). 18S RNA probe was used as loading control

To examine which L-arabinose-induced genes are expressed in the various mutants during exponential and stationary phase, the expression of several AraR target genes *abfA*, *abfB*, *abfC*, *abnA*, and *ladA* were monitored at time points representing exponential phase (90% of maximal biomass accumulation) and time point representing stationary phase (20 h after D-fructose depletion) (Fig. 3).

The expression of arabinofuranosidases *abfA*, *abfB*, and *abfC* correlated well with the arabinofuranosidase activity measurements. No expression of *abfA*, *abfB*, or *abfC* was detected during exponential growth in any of the strains that contain a functional *creA* gene. Also *abnA* and *ladA* were not expressed during exponential growth. Strains containing the AraR^N806I^ allele (JR13.2 and EH3.1) showed expression of the AraR target genes *abnA*, *abfB*, and *abfC* only during stationary phase (Fig. 3). This suggests that expression of these genes by the constitutive AraR^N806I^ occurs only under derepressing conditions which fully supports the arabinofuranosidase activity measurements. Combining the constitutive AraR^N806I^ allele together with the deletion of *creA* (JN16.1#7) resulted in expression of all the AraR target genes investigated both during exponential and stationary phases (Fig. 3). Expression levels of *ladA* and *abfA* show similar patterns in that they are moderately expressed during exponential growth in the *araR*^*N806I*^*ΔcreA* mutant and low during starvation. Expression levels of *abfB* and *abfC* are relatively high both during exponential phase and stationary phase in the *araR*^*N806I*^*ΔcreA* mutant, and *abnA* is only expressed in the *araR*^*N806I*^*ΔcreA* mutant during stationary phase. This complex pattern of regulation suggests that the expression of different genes that are part of the AraR regulon are also affected by other factors, such as CreA.

## Discussion

The expression of genes encoding arabinolytic enzymes is tightly controlled to ensure that enzyme production only occurs in the presence of arabinan or its derivatives like L-arabinose or L-arabitol. In *Eurotiomycetes*, the expression of genes encoding arabinolytic enzymes is controlled by the Zn(II)_2_Cys_6_ transcriptional activator AraR (Battaglia et al. 2011, 2014; Gruben et al. 2017). Arabinolytic enzymes are not only subjected to induction by an inducer, they are also repression control via the CreA-mediated carbon catabolite control (Fig. 1b; van der Veen et al. 1993, 1994; Flipphi et al. 1994). Using the power of a positive selection strategy, we were able to identify several mutations in the AraR transcriptional regulator that lead to constitutive expression of AraR-controlled genes. The screen for mutants was performed in a *ΔcreA* background to uncouple the activation and repression mechanisms. Subsequent analysis of the inducer-independent AraR^N806I^ mutant revealed that the inducer-independent expression was still sensitive to CreA-mediated repression. Additional attempts to isolate inducer-independent mutants in a non-*creA* background were not successful (Reijngoud and Ram), indicating that in our screen, is it difficult to obtain mutants that bypass the repression of CreA.

All mutations that were found in AraR are missense mutations. No frameshift or non-sense mutations leading to truncated proteins were identified. This indirectly implies there is probably no repressor protein involved in the regulation of L-arabinose-induced genes that upon loss of function results in a constitutive expression of these genes as has been found before for the regulation of galacturonic-induced genes (Niu et al. 2017). If a repressor protein would be involved, it is reasonable to assume that repressor mutants would be picked up at high frequency, as loss of function mutations in the repressor are likely to occur more frequently compared to gain of function mutations in AraR. Interestingly, we identified one constitutive mutant which did not have a mutation in AraR or XlnR and this could represent another factor involved in controlling gene expression. Molecular genetic analysis of this mutant by genome sequencing is currently performed, but outside the scope of this study.

AraR shows significant sequence similarities to its paralog XlnR which controls the expression of genes encoding xylanolytic enzymes (Battaglia et al. 2011). In *Aspergillus* species, it has been shown that AraR and XlnR have overlapping functions (Battaglia et al. 2011, 2014; Ishikawa et al. 2018) and it has recently been shown that XlnR and AraR of *A. oryzae* bind to similar DNA-binding sequences in their target genes (Ishikawa et al. 2018). Transcriptomic and DNA-binding studies in *A. niger* and *A. oryzae* have shown that some genes are specifically induced via AraR, others by XlnR, and again others are induced by both XlnR and AraR (Gruben et al. 2017; Ishikawa et al. 2018).

The *abfA* gene has been reported to be specifically induced by arabinan, L-arabinose, and L-arabitol (Flipphi et al. 1994; Kowalczyk et al. 2017; Gruben et al. 2017) which was also confirmed using our reporter strain (Fig. 1a, upper row). However, when *creA* was deleted in the *PabfA-amdS* reporter strain, the expression of *abfA* was also induced by xylan, D-xylose, and xylitol (Fig. 1a, lower row). This observation suggests that the activated XlnR transcription factor can also induce expression of primary AraR target genes, but only when CreA is absent. It suggests competition for binding sites of the different transcription factors and that the absence of CreA might affect the binding affinity of XlnR to the *abfA* promoter resulting in higher expression of *abfA* on D-xylose. Alternatively, XlnR levels might be increased in the *ΔcreA* strain and the higher abundance of XlnR might allow binding even when the binding affinity is not very high. The *abfA* gene contains two putative XlnR/AraR binding sites at − 383 and − 94 relative to the start codon. The binding site at position − 383 (CGGCTAAA) matches the binding site predicted to have preferred binding of XlnR while the binding site at position − 94 (CGGTTAAT) is predicted to bind both XlnR and AraR (Ishikawa et al. 2018). The 1000-bp promoter region of *abfA* contains 14 putative CreA binding sites (SYGGRG), one of which at position − 97 which overlaps with the putative XlnR/AraR binding site. It should be noted that the XlnR/AraR or XlnR-specific binding sites are based on studies in *A. oryzae* (Ishikawa et al. 2018) and a similar sequence specificity has not been shown for *A. niger*.

Our results also indicate possible competition of inactive and active AraR for DNA binding, because of the observed semi-dominant effect when expressing constitutively active AraR in a wild-type AraR background. This observation suggests that under non-inducing conditions, inactive AraR might bind the AraR binding site in the promoter region and compete with the activated form of AraR (AraR^N806I^). Kowalczyk et al. (2015) described an interesting observation that genes encoding arabinolytic enzymes are higher expressed in response to L-arabinose in a *ΔxlnR* strain. As XlnR and AraR have very similar binding sites (Ishikawa et al. 2018), this result can be interpreted such that also inactive XlnR binds to the same binding site and competes with active AraR, explaining the observation that deletion of XlnR results in higher expression of arabinases.

In both *A. niger* and in *Trichoderma reesei*, specific point mutations in XlnR and Xyr1 have been described that lead to constitutive production and expression of xylanases. The two mutations are the XlnR^V756F^ mutation in *A. niger* and the Xyr1^A824V^ mutation in *T. reesei* (Hasper et al. 2004; Derntl et al. 2013). In protein sequence alignment of XlnR and Xyr1, these two mutations are only three amino acids apart from each other (Supplemental Fig. S1). The C-terminal domain of XlnR/Xyr1 including these mutations has been suggested to be an activator domain (Hasper et al. 2004). Mutations in AraR leading to constitutive expression were not limited to a specific domain or well-conserved region in AraR. The alleles leading to the highest AraF activity (mutation at position 756 or 806) were found in the C-terminal part of the protein, indicating that this domain might serve as the AraR activation domain, similar as has been suggested for XlnR.

ClustalW alignment of XlnR/Xyr1 and AraR protein sequences from *A. niger*, *A. nidulans*, *A. fumigatus*, and *A. oryzae* and Xyr1 of *T. reesei* was performed to analyse whether the mutated amino acids in *A. niger* AraR were conserved among AraR or XlnR orthologs. From the nine amino acid changes in AraR that lead to constitutive expression, eight amino acids were fully conserved among AraR orthologs. Only the serine at position 675 in *A. niger* was not conserved in *A. fumigatus* (Table 3; Supplemental Fig. S5). Mutations resulting in amino acid changes at positions 504, 600, and 809 in AraR protein of *A. niger* were conserved in both AraR and XlnR/Xyr1. The valine at position 756 in XlnR of *A. niger* that upon change to phenylalanine leads to constitutive activation of xylanases (Hasper et al. 2004) is conserved among Aspergilli and *T. reesei* XlnR orthologs, but not in AraR homologs. The alanine at position 824 in *T. reesei* that upon change to valine leads to constitutive activation in *T. reesei* Xyr1 is conserved among all XlnR and AraR homologs (Supplemental Fig. S1). In a very recent paper, Gao et al. (2019) showed that overexpression of the homologous alanine to valine mutation in AraR of *Penicillium oxalicum* (AraR^A731V^) results in constitutive α-L-arabinofuranosidase activity under carbon starvation conditions also in this species). However, our results show that also mutations in several other positions in the AraR protein can result in (partial) constitutively. It will therefore be of interest to examine whether these additional mutations can be introduced in AraR or XlnR other species to increase arabinolytic and/or xylanolytic enzyme production. The newly identified amino acid changes leading to inducer-independent expression presented in this study also open the possibility to combine individual mutations to examine whether these mutations have additive or possible synergistic effects on enzyme production.

**Table 2:** Growth characteristics and arabinofuranosidase activities of *A. niger* strains in pH-controlled bioreactors

Strain	Genotype	Maximum biomass (g/kg)	μmax (h^−1^)	Arabinofuranosidase activity* (nmol PNP/ml/min)			
				70% biomass	90% biomass	20 h after D-fructose depletion	70 h after D-fructose depletion
JR12.3	*araR*^*wt*^ + *araR*^*wt*^ (pyrG**)	4.29 ± 0.01	0.21 ± 0.00	< 0.1	< 0.1	10.9 ± 0.7	11.9 ± 0.1
JR13.9	*araR*^*wt*^ + *araR*^*N806I*^ (pyrG**)	4.34 ± 0.03	0.21 ± 0.01	< 0.1	< 0.1	66.7 ± 1.4	103.1 ± 3.4
JR13.2	*araR*^*wt*^ + *araR*^*N806I*^ (pyrG**) + mc *araR*^*N806I*^	4.39 ± 0.01	0.22 ± 0.00	< 0.1	< 0.1	322.3 ± 5.6	Not determined
EH3.1	*ΔaraR araR*^*N806I*^ (pyrG**)	4.40 ± 0.02	0.20 ± 0.01	< 0.1	< 0.1	182.6 ± 3.3	255.7 ± 7.7
JN16.1	*araR* ^*wt*^ *ΔcreA::hygB*	4.35 ± 0.03	0.15 ± 0.02	< 0.1	< 0.1	4.2 ± 0.2	6.7 ± 0.3
JN16.1 U7	*araR* ^*N806I*^ *ΔcreA::hygB*	4.60 ± 0.02	0.16 ± 0.00	46.8 ± 2.5	58.61 ± 2.0	219.1 ± 11.4	288.8 ± 1.2

*Total arabinofuranosidase activity when grown in submerged 5 l batch cultivation with 0.75% D-fructose and 0.0003% yeast extract. Maximum biomass and time point of carbon deletion are determined using offline biomass measurements at the corresponding time points. Standard variation between the biological duplicates are given

**Table 3:** Conservation of mutated amino acids in constitutive AraR variants of *A. niger* (Anig) in AraR and XlnR of *A. niger*, *Aspergillus nidulans* (Anid), *Aspergillus fumigatus* (Afum), and *Aspergillus oryzae* (Aory) and Xyr1 of *T. reesei*

Position amino acid mutated	Anig AraR (A2QJX5)	Afum AraR (OXN19303)	Anid AraR (Q5BGE2)	Aory AraR (XP_023090294)	Anig XlnR (CAK42534)	Afum XlnR (B0XUL1)	Anid XlnR (CAC81360)	Aory XlnR (Q2UD93)	*T. reesei* Xyr1 (AAO33577)
R307L	**R**	**R**	**R**	**R**	D	D	E	D	E
I399M	**I**	**I**	**I**	**I**	I	V	V	I	V
F416Y	**F**	**F**	**F**	**F**	W	W	W	W	W
D504N	*D*	*D*	*D*	*D*	*D*	*D*	*D*	*D*	*D*
G600E	*G*	*G*	*G*	*G*	*G*	*G*	*G*	*G*	*G*
S671 L	S	A	S	S	A	A	A	A	A
I763F	**I**	**I**	**I**	**I**	V	V	V	V	V
N806I	**N**	**N**	**N**	**N**	D	D	D	D	D
E809K	*E*	*E*	*E*	*E*	*E*	*E*	*E*	*E*	*E*

Amino acid residues in italics are conserved in both AraR and XlnR, and amino acids in bold only in AraR. Accession numbers of the proteins are given within brackets below the gene name

Little is known about the mechanism by which AraR is activated. Earlier studies have shown that intracellular L-arabitol is the inducer to activate the expression of genes encoding arabinolytic enzymes, because when the intracellular concentration of L-arabitol is high, for example, in a D-xylulose kinase defective mutant, a high arabinase production is observed (Witteveen et al. 1989; de Vries et al. 1994). Whether AraR directly interacts with the inducer resulting in its activation or whether the presence of L-arabitol induces post-translational modifications resulting in AraR activation is unknown. As mentioned above, AraR is highly similar in sequence to XlnR, the transcriptional activator of xylanolytic genes. The exact activation mechanism for XlnR is unknown, but phosphate-affinity SDS-PAGE analysis of XlnR in *A. oryzae* has shown that the C-terminal half of XlnR is highly phosphorylated when D-xylose is present, indicating that phosphorylation controls XlnR activity (Noguchi et al. 2011). Which residues in the C-terminus are phosphorylated and whether the phosphorylation is important for XlnR activation is unknown. Whether the AraR phosphorylation status also differs between inducing and non-inducing conditions awaits further studies.

## Electronic supplementary material

ESM 1 (PDF 663 kb)
