# Supplementary material for: Mutations in AraR leading to constitutive of arabinolytic genes in Aspergillus niger under derepressing conditions
Source: Appl Microbiol Biotechnol. 2019 Apr 8;103(10):4125–36. doi: 10.1007/s00253-019-09777-0 (PMC6486530; doi:10.1007/s00253-019-09777-0)
Supplement: Supplementary file 1 — (PDF 663 kb) [file 253_2019_9777_MOESM1_ESM.pdf]

Supplemental figures/tables file

Applied Microbiology and Biotechnology

Mutations in AraR leading to constitutive expression of arabinolytic genes in *Aspergillus niger* under derepressing conditions

Jos Reijngoud[1], Malte Deseke[1], Elmar T.M. Halbesma[1], Ebru Alazi[1*], Mark Arentshorst[1], Peter J. Punt[1,2], Arthur F.J. Ram[1]**

[1]Molecular Microbiology and Biotechnology, Institute of Biology Leiden, Leiden University, The Netherlands

[2]Dutch DNA Biotech, Hugo R Kruytgebouw 4-Noord, Padualaan 8, 3584 CH Utrecht, The Netherlands.

* current address: Dutch DNA Biotech, Hugo R Kruytgebouw 4-Noord, Padualaan 8, 3584 CH Utrecht, The Netherlands

**corresponding author

Corresponding Author: E-mail: a.f.j.ram@biology.leidenuniv.nl, Tel: +31 71 527 4914; Fax: +31 71 527 4900

Supplemental files

Supplemental Table 1. Overview of the used primers

Supplemental Fig. 1. Southern blots analysis of *A. niger* strains EH1.2

Supplemental Fig. 2. Southern blots analysis of *A. niger* strains EH2.1 and EH3.1

Supplemental Fig. 3. Diagnostic PCR analysis of *A. niger creA* deletion strains JR16.2 and JR17.2

Supplemental Fig. 4. Southern blots analysis of *A. niger* strains JR12.3, 13.2 and 13.9

Supplemental Fig. 5. Alignments AraR and XlnR in four *Aspergillus* species and XlnR of *Trichoderma reesei*

Supplemental Table 1. Overview of the primers used in this study

Primer name	Sequence (5'-3')	Remark	Used for
araRP1f	ACCGTTTGGAAAGTAGTGG		Amplification and sequencing *araR*
araRP2r	AAGCGGGGAGAAAGAGACCG		Sequencing *araR*
araRP3f	TGGATCGCTTCTGCTCTGCC		Sequencing *araR*
araRP4r	TGTCGCATTAACAGTGTGCC		Sequencing *araR*
araRP5f	AGGACGCATTGCGCGATCCC		Sequencing *araR*
araRP6r	CGACCGCAGCATGAAGTGGG		Sequencing *araR*
araRP7f	CTCCTCGACCTAGCAGCC		Sequencing *araR*
araRP8r	TTCTCGCCTGACAGTGGG		Sequencing *araR*
araRP9f	TTGCGTATGCGACCGTGGCC		Sequencing *araR*
araRP10r	CCCGCTTCTTTCTGCAGCCG		Sequencing *araR*
araRP11f	TCGAGGATAAGGACTTCTGG		Sequencing *araR*
araRP12r	CGATCCAAAACAAACAAGCC		Sequencing *araR*
araRP13f	CTCGGTTGATTATTTGCC		Sequencing *araR*
araRP14r	TGTCGCTGTAGCTGGAGG		Amplification and sequencing *araR*
araRP1f	ACCGTTTGGAAAGTAGTGG		*ΔaraR* Bipartite fragment - 5' *araR* flank::5' *nicB*
araRP18r	**CAATTCCAGCAGCGGCTT** GCCGACATTGGCGATTTC	Overlap in bold	*ΔaraR* Bipartite fragment - 5' *araR* flank::5' *nicB*
nicBnidP5f	**AAGCCGCTGCTGGAATTG** CGTTATGCACAGCTCCGTCTT	Overlap in bold	*ΔaraR* Bipartite fragment - 5' *araR* flank::5' *nicB*
nicBnidP8r	GAACAGCCTTCGGGATTGC		*ΔaraR* Bipartite fragment - 5' *araR* flank::5' *nicB*
nicBnidP7f	CGCCTTATATCCGATTGGCTT		*ΔaraR* Bipartite fragment - 3' *nicB*::3' *araR* flank
nicBnidP6r	**CGATGGATAATTGTGCCGTGT**GCGCATACACAGAAGCATTGA	Overlap in bold	*ΔaraR* Bipartite fragment - 3' *nicB*::3' *araR* flank
araRP17f	**ACACGGCACAATTATCCATCG** AGGCCATCGGTTGGAGAGA	Overlap in bold	*ΔaraR* Bipartite fragment - 3' *nicB*::3' *araR* flank
araRP14r	TGTCGCTGTAGCTGGAGG		*ΔaraR* Bipartite fragment - 3' *nicB*::3' *araR* flank
araRP1f	ACCGTTTGGAAAGTAGTGG		Southern blot probe - verification *araR* knock out EH1.2 (5' flank)
araRP18r	CAATTCCAGCAGCGGCTT GCCGACATTGGCGATTTC		Southern blot probe - verification *araR* knock out EH1.2 (5' flank)

araRP17f	ACACGGCACAATTATCCATCG AGGCCATCGGTTGGAGAGA		Southern blot probe - verification *araR* knock out EH1.2 (3' flank)
araRP14r	TGTCGCTGTAGCTGGAGG		Southern blot probe - verification *araR* knock out EH1.2 (3' flank)
araRP15f-NotI	aaggaaaaaaGCGGCCGCACCGTTTGGAAAGTAGTGG	*Not*I underlined	PCR amplification araR allele 1kb up for cloning in MA334
araRP16r-NotI	aaggaaaaaaGCGGCCGCTGTCGCTGTAGCTGGAGG	*Not*I underlined	PCR amplification araR allele 1kb down for cloning in MA334
ABpyrGP12for-EcoRI-AscI	cggaattcggcgcgccCGGC TGACGTTACCACCACT	*Eco*RI, *Asc*I underlined	Southern blot probe - verification of *pyrG-araR* integration (5' flank)
ABpyrGP10rev-NotI	aaggaaaaaaGCGGCCGCAGTCAGACCTAATGCCTCGGG	*Not*I underlined	Southern blot probe - verification of *pyrG-araR* integration (5' flank)
ABpyrGP11for-NotI	aaggaaaaaaGCGGCCGC CGTCGCGTGATAAGGGTTG	*Not*I underlined	Southern blot probe - verification of *pyrG-araR* integration (3' flank)
ABpyrGP13rev-EcoRI-AscI	cggaattcggcgcgccTCGGGTCAATTTCCTCTGTTG	*Eco*RI, *Asc*I underlined	Southern blot probe - verification of *pyrG-araR* integration (3' flank)
abfA_f	AATCACTCAGGAGATGGAGG		*abfA* probe
abfA_r	TTGACTTATAGGTTTCGCC		
AnAbfBP1f	GTTCACGTGGGCCCCTGTGACATCTACGAAG		*abfB probe*
AnAbfBP2r	CGTCTAGAAACGAAGCAAACGCCGTCTCAATC		
abfCP1f	TTGAAGCTCCTTGATCCCAG		*abfC* probe
abfCP2r	ATGGTCCATCCCCGCATATA		
abnA_f	GCCCCGGATGTCTCC		*abnA probe*
abnA_r	GAAATCACCCGTGGGCG		
18SrRNANF	GCCTGCGGCTTAATTTGACTC		18S probe
18SrRNANR	TGACCAACTTTCCGGCTCTG		

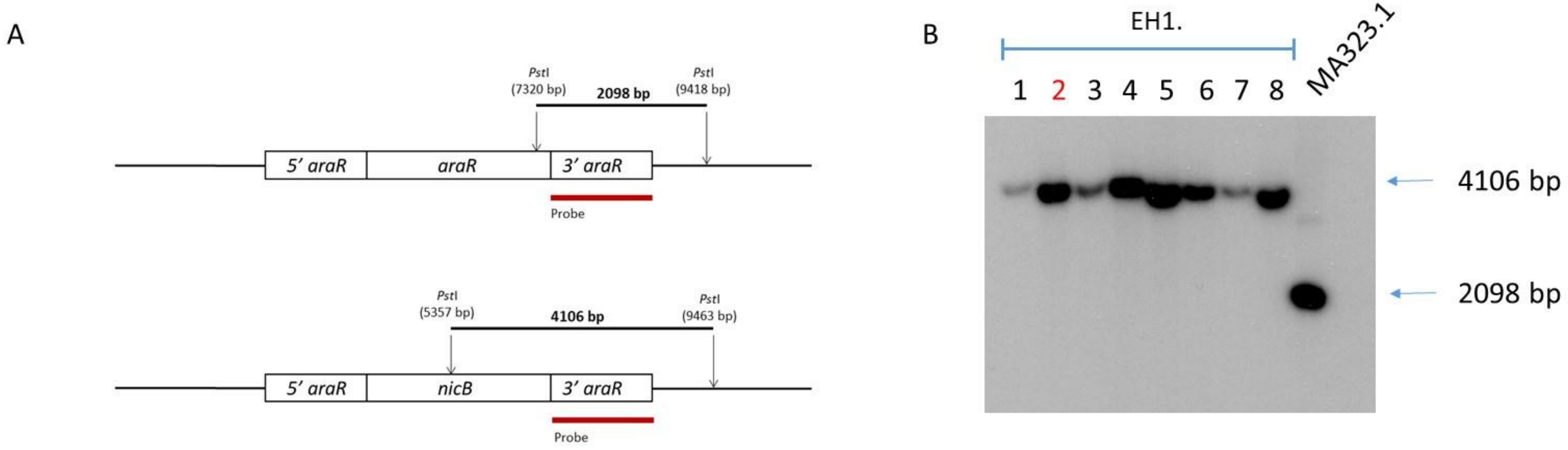

Supplemental Fig. 1. Southern blot analysis to verify *araR::nicB* deletion in MA323.1 to give EH1.2. A) Schematic representation of *araR* locus and in the *araR::nicB* locus in the deletion strain. DNA fragments expected to hybridize after digestion of genomic DNA with *Pst*I are indicated. B) Genomic DNA of eight putative *araR::hygB* transformants (EH1.1 to EH1.8) and the parental strain (MA323.1) was analysed. EH1.2 was selected for further analysis.

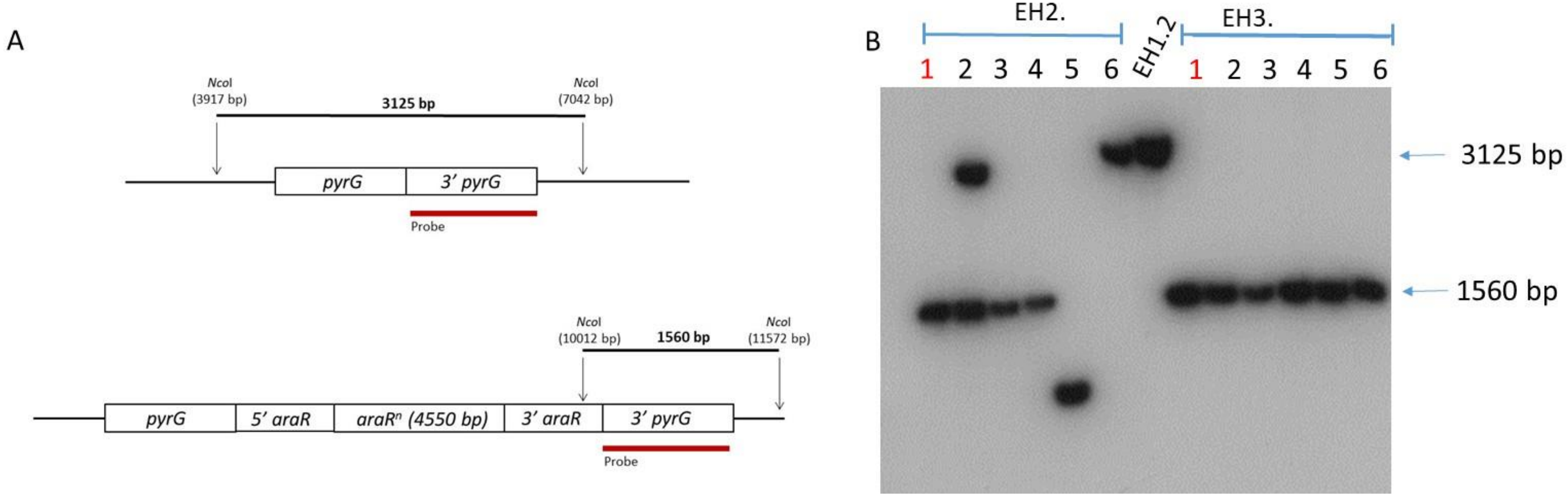

Supplemental Fig. 2. Southern blot analysis to verify *araR* integration in EH1.2 to give EH2.1 and EH3.1. A) Schematic representation of *pyrG* locus in EH1.2 and after integration of the *araR* gene at the *pyrG* locus. DNA fragments expected to hybridize after digestion of genomic DNA with *Nco*I are indicated. B) Genomic DNA of six putative *araR*^{wt} and *araR*^{N801I} transformants (EH2.1 to EH2.6 and EH3.1 to EH3.6 and the parental strain (EH1.2) was analysed. EH2.1 and EH3.1 (indicated in red) were selected for further analysis.

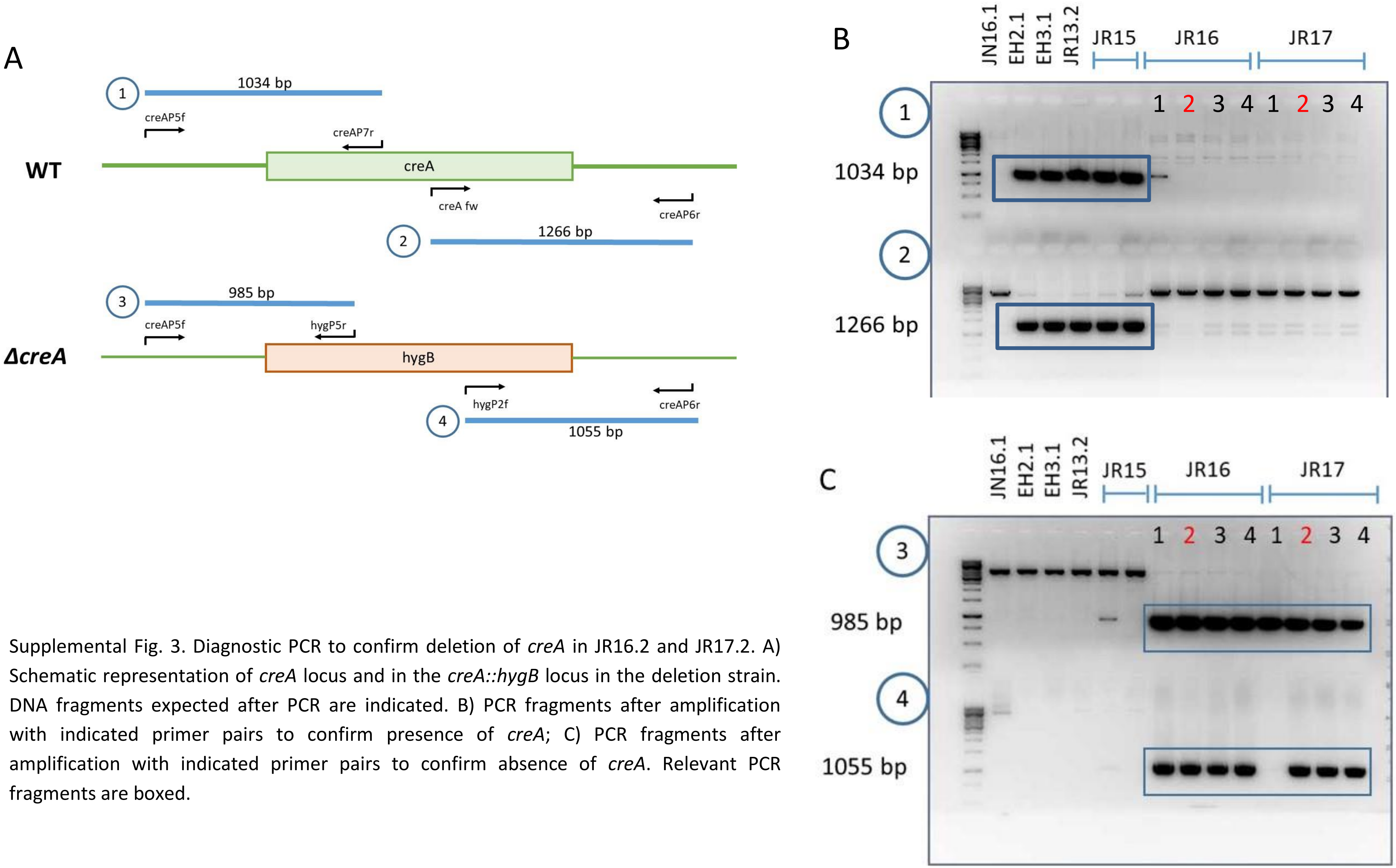

Supplemental Fig. 3. Diagnostic PCR to confirm deletion of *creA* in JR16.2 and JR17.2. A) Schematic representation of *creA* locus and in the *creA::hygB* locus in the deletion strain. DNA fragments expected after PCR are indicated. B) PCR fragments after amplification with indicated primer pairs to confirm presence of *creA*; C) PCR fragments after amplification with indicated primer pairs to confirm absence of *creA*. Relevant PCR fragments are boxed.

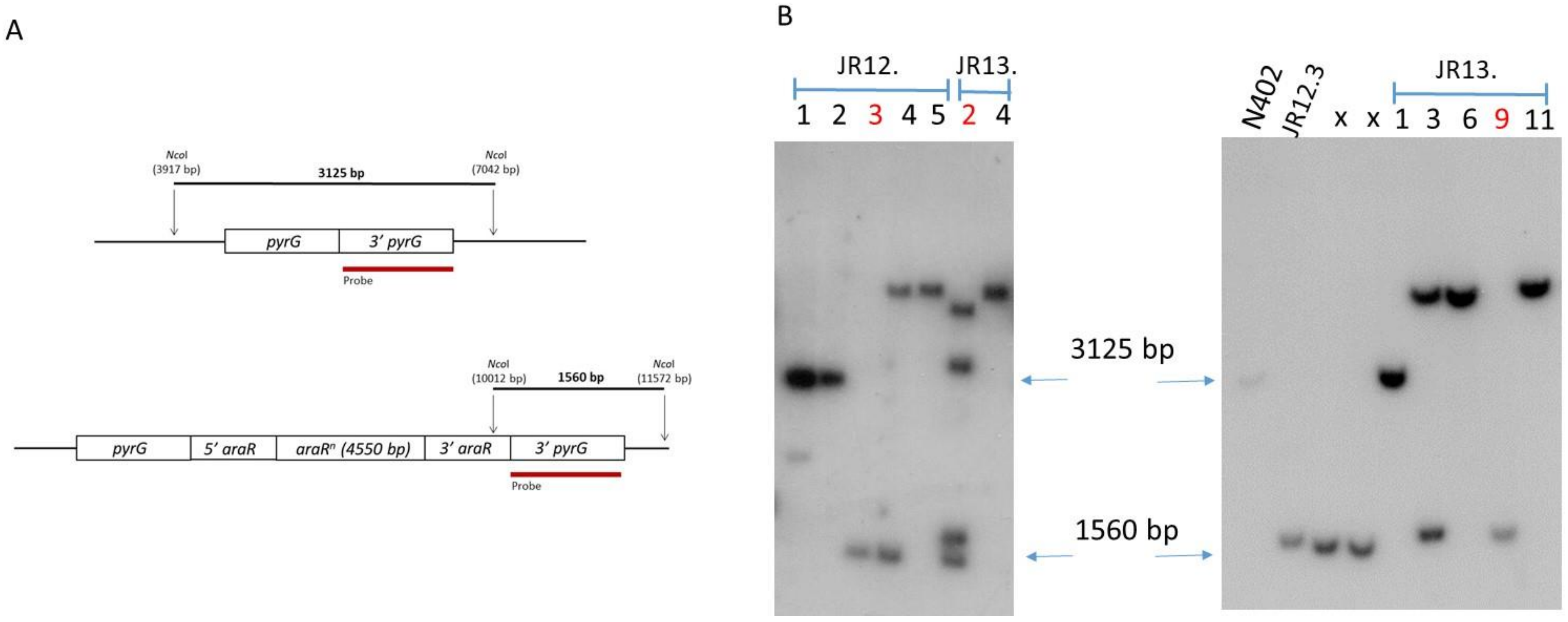

Supplemental Fig. 4. Southern blot analysis to verify *araR*^{wt} or *araR*^{N801I} integration in AB4.1 to give JR12.3, JR13.2 and JR13.9 A) Schematic representation of *pyrG* locus in AB4.1 and after integration of the *araR* gene at the *pyrG* locus. DNA fragments expected to hybridize after digestion of genomic DNA with *Nco*I are indicated. B) Genomic DNA of pattern of transformants. JR12.3 single-copy *araR*^{wt}, JR13.2 (multi-copy *araR*^{N801I}) and JR13.9 (single-copy *araR*^{N801I}) (indicated in red) were selected for further analysis.

Supplemental Fig. 5. Alignments AraR and XlnR in four Aspergilli species and Xyr1 from *Trichoderma reesei*

```
AnidAraR(1-825)      ------------------------------------------------------------   0
AoryAraR(1-830)      ------------------------------------------------------------   0
AfumAraR(1-838)      ------------------------------------------------------------   0
AnigAraR(1-832)      ------------------------------------------------------------   0
TreXyr1(1-940)       MLSNPLRRYSAYPDISSASFDPNYHG----SQSHLHSINVNTFGNSHPYPMQHLAQHAEL   56
AnidXlnR(1-875)      -----------------------------MSQSQSQTIGLDTLAEGSQYVLEQLQLSREG   31
AoryXlnR(1-971)      MSTTSIQHFT-------SSFSPFSSGTQPVGMAQSQTVGLDTLAEGSQYALEQLQLSREA   53
AnigXlnR(1-875)      ------------------------------------------------------------   0
AfumXlnR             MSTTSLQHFP-------HSYSPFSSSRSLNRMAQSQTSGLDTLAEGSQYALEQLQMSREA   53


AnidAraR(1-825)      ------------------------------MASSH-----QGN---G---TVPNSQTDAP   19
AoryAraR(1-830)      ------------------------------MDPAQ-----SGD---APQGNTQGTTDD-P   21
AfumAraR(1-838)      ------------------------------MDLAQ-----PGD---APPTSTNTAGTLED   22
AnigAraR(1-832)      ------------------------------MDSAQ-----SGD---AQAPNVPAATEEPT   22
TreXyr1(1-940)       SSSRM--------I------------------------RASPVQPKQRQGSLIAAR--KN   82
AnidXlnR(1-875)      GNSE----------------------------------NNSTFKPSSVRDSLAEARSMIR   57
AoryXlnR(1-971)      NGASAVDGGVPNPLRSSISKPQGQQLYSDESSAQHTQNATTGFRNLPQRDQLAEARSTIR   113
AnigXlnR(1-875)      ---------MSHT--------KDQ--PP--FDNEKNQSTGSGFRDALQRDPLVEARSAIR   39
AfumXlnR             AGSGEATDSVGKP--------KDQ--FQVDNDNHHNNHSLSNFKNPSQRDPLVEARSTIR   103
                                                                           :

AnidAraR(1-825)      PDSSTKRRWRRNRIACDSCHARRVRCDRQFPCSRCLRSEITCEFTRERRKRGRIARSKLA   79
AoryAraR(1-830)      QNSSAKRKWRRNRIACDACHARRVRCDRAFPCSRCLRSEIQCEFTRERRKRGRIARSKQT   81
AfumAraR(1-838)      SSSTAKRRWRRNRIACDSCHSRRVRCDRAFPCSRCLRSEIRCEFTRERRKRGRIARSKQT   82
AnigAraR(1-832)      GEATTKRRWRRNRIACDSCHSRRVRCDRAFPCSRCLRSDIRCEFTRERRKRGRIARSRLV   82
TreXyr1(1-940)       STGTAGPIRRRISRACDQCNQLRTKCDGLHPCAHCIEFGLGCEYVRERKKRGKASRKDIA   142
AnidXlnR(1-875)      KNSSSAPVRRRISRACDQCNQLRTKCDGQNPCAHCIEFGLTCEYARERKKRGKASKKDIA   117
AoryXlnR(1-971)      KSSNSGPVRRRISRACDQCNQLRTKCDGQNPCAHCIEFGLTCEYARERKKRGKASKKDLA   173
AnigXlnR(1-875)      KTSSSAPVRRRISRACDQCNQLRTKCDGQHPCAHCIEFGLTCEYARERKKRGKASKKDLA   99
AfumXlnR             KNSASAPVRRRISRACDQCNQLRTKCDGQNPCAHCIDFGLTCEYARERKKRGKASKKDLA   163
                       . :    **   *** *:  *.:**   **::*:   : **:.***:***: ::.  .
```

```
AnidAraR(1-825)       EMAKNKMETSETPAPAKT------------MNGIP-----APAGTEIPGHVSPASTFHHR   122
AoryAraR(1-830)       NTNGGTEKLSAALSNGRV------------PAPSTADAGGDAVATPAPN-GSPSSSFPHR   128
AfumAraR(1-838)       ATVPNGGSMEKLPKAPNV------------QPPVPAAVPADAAPTPLPNHASPTTSFQHR   130
AnigAraR(1-832)       ETKTA---VEKASQPV--------------ETRDSAPAPAEAGSGPVPN-GSPSSTFHHR   124
TreXyr1(1-940)        AQQAAAAAAQHSGQVQDGPEDQHRK--LSRQQSESSRGSAEL---AQPAHDPPHGHIEGS   197
AnidXlnR(1-875)       AAAAAAGHQG----------------GMGNRSPTDRRLSQEPGGRYDSVLEAS-----RV   156
AoryXlnR(1-971)       AAAAAVANNGTA-PTSNGNTSNDSVSSAKRHTPSDGQSTQEVSGRYDPNFDAS-----RN   227
AnigXlnR(1-875)       AAAAAAT------QGSNGHSGQANASLMGERTSEDSRPGQDVNGTYDSAFESH-----HL   148
AfumXlnR              AAAAAAAAAATNSGQPNGSSGKEDAALVGGHTSPDRRP--TINGRYDPAFEVP-----RN   216

AnidAraR(1-825)       SPP-----------ANA----------PTVSAPSVDGRRSQTDPQLP------------V   149
AoryAraR(1-830)       SPG-----------TNE----------MTASAPSVDERRSQAELPLA------------P   155
AfumAraR(1-838)       SPA-----------TNE----------MTVSAHSIDDRRSQADPSLP------------A   157
AnigAraR(1-832)       SPA-----------TND----------VTGSAPSIDERRSQADVSLP------------P   151
TreXyr1(1-940)        VSSFSDNGLSQHAAMGGMDGLEDHHGH-VGVDPALGRTQLEASSAMGLGAYGEVH----P   252
AnidXlnR(1-875)       Q---------SHLPANGLSSIHNTQ--AAHSQPPLGSA----LDALHLNHFTQLNESGRS   201
AoryXlnR(1-971)       LATAGQSQLGQHSDMSGMAGMQGSQQ-TPHSQPSLGGA----IDAIHLNHFNTLNDSNRP   282
AnigXlnR(1-875)       SSQPSHM---QHASTAGISGLHESQTAPSHSQPSLGTT----IDAMHLNHFNTMNDSGRP   201
AfumXlnR              L--NGSA---QHSEASGMVGMQNSQHLPPHSQSSMGGG----LEGLPLNGYNGLNDSGRP   267
                                                        :.         :

AnidAraR(1-825)       RRPEIGGNVTEEWLAGTH---VSPGSYEFLNGPAFGEGLGPF----------PHMFDVWN   196
AoryAraR(1-830)       RKLAPGGNVTEEWLAAAH---VSPGSYDFLGG-GLG--EGPF----------PRMFDVWS   199
AfumAraR(1-838)       RRPGPTGNVTEEWLSAAH---VSPDSYEVLGGGAWG--DGPL----------PRVLDIWN   202
AnigAraR(1-832)       RKPGHTVNATEEWLAGTH---VSPGSYEPLAGIGPG--EGPF----------PRIFDIWN   196
TreXyr1(1-940)        GYESPGMNG-HVMV---------PPS-YGAQTTMAGYSGISYAAQAPSPATYSSDGNFRL   301
AnidXlnR(1-875)       QMPVSDLRS-LQILHNNPR---SPSA-L-PHGL-NAYNDNTFSLLNSQEPNTTSLNHFRL   254
AoryXlnR(1-971)       QMSVPDLRS-LQMLHPSGANTRSPSGALPPQGMNSGYNDGAYSLMNASEANHPSINQYRL   341
AnigXlnR(1-875)       AMSISDLRS-L------------PPSVLPPQGLSSGYNASAFALVNPQEPGSP-ANQFRL   247
AfumXlnR              SMPVPELQS-LHMLHNSHTNPRSPSSILPHHRYNGGYNDSAYSLMNPQEPNSTSISHFRL   326
                             .               * .         .                    .
```

```
AnidAraR(1-825)      GVDLAAYSAG----TSQGSKAT-------------N---APSTSTAPLKYPVLQPLMPFV   236
AoryAraR(1-830)      GVDLSNNSGP----TPQGAKVS-------------GAGQTPGVPPASLKYPVLQPLMPFL   242
AfumAraR(1-838)      GADLAGYSAP----TVQSSKPA-------------GAARAPSISSTTLKYPVLQPLMPFL   245
AnigAraR(1-832)      GVDLAGYSDP----ASQGSKIT-------------GLGQTPAPSATILKYPVLQPVMPYL   239
TreXyr1(1-940)       TGHIHDYPL------ANGSSPSWGVSLASPSNQFQLQLSQPIFKQSDLRYPVLEPLLPHL   355
AnidXlnR(1-875)      GNSTDNPSAQFLGLSPPAQSPGWL-PLPSPSPANFPSFPMAPFSGTSLRYPVLQPVLPHI   313
AoryXlnR(1-971)      GNSAENPPAPFLGLSPPAQSPGWL-SLPSPSPANFASFSMPPFSS-TLRYPVLQPVLPHI   399
AnigXlnR(1-875)      GSSAENPTAPFLGLSPPGQSPGWL-PLPSPSPANFPSFSLHPFSS-TLRYPVLQPVLPHI   305
AfumXlnR             GSSTENPPNSFLGLSPPAQSPGWL-PLPSPSPANFPSFSMASFST-TLRYPVLHPVLPHI   384
                                      . .                            *:****.*::*.:

AnidAraR(1-825)      EATLPRKLVFDLLDLYFTSAFSTHMHPV------------SFLSKDAPRPSSPALLSSML   284
AoryAraR(1-830)      DANLPRRLVFDLLELYFTSAFSTQMHPVCHHIHSYILRKASFLSRDAPRPSTPALLASML   302
AfumAraR(1-838)      EANLPRRLVFDLLELYFTSAFSTHMHPVCHHIHCYVLRKASFLSRENPRPSSPALLASML   305
AnigAraR(1-832)      ESSLPRKLVYDLLDLYFTSAFSTHMHPVCHHIHCYVLRKASFLSREAPRPSSPALLASML   299
TreXyr1(1-940)       GNILPVSLACDLIDLYFSSSSSAQMHPMSPYVLGFVFRKRSFLHPTNPRRCQPALLASML   415
AnidXlnR(1-875)      ASIIPQSLACDLLDLYFTSSSSSHLSPQSPYVVGYIFRKQSFLHPTKPRVCSPGLLASML   373
AoryXlnR(1-971)      ASIIPQSLACDLLDVYFTSFSPSHLSPQSPYVVGYIFRKQSFLHPTKPRVCSPGLLASML   459
AnigXlnR(1-875)      ASIIPQSLACDLLDVYFTSSSSSHLSPLSPYVVGYIFRKQSFLHPTKPRICSPGLLASML   365
AfumXlnR             ASIIPQSLACDLLDVYFTSSSSSHLSPQSPYVVGYIFRKQSFLHPTKPRVCTPGLLASML   444
                        :*  *. **:::**:*   ::: *             ***    ** . *.**:***
                       AraR-307
AnidAraR(1-825)      WVAALDDRAFSLPISPPQRKRICQFLCALTIRLLRPLIHVSFKDQGGAAAAVAAAAAAAT   344
AoryAraR(1-830)      WVAALDDRAFALSISPPQQRKNICQFLCALTIRLLRPLIHVSFKDQGAS------------   350
AfumAraR(1-838)      WVAALDDRAFALSISPPQRKKICQFLCALTIRLLRPLIHVSFKEQAGSNA----------   355
AnigAraR(1-832)      WVAALDDRAFALPISPPQRKKICQFLCALTLRLLRPLIHVSFKEQEGAAA----------   349
TreXyr1(1-940)       WVAAQTSEASFLTSLPSARSKVCQKLLELTVGLLQPLIHTG---TNSPSPKTSP----VV   468
AnidXlnR(1-875)      WVGAQTSDAPFLTSPPSARGRVCQKLLELTIGLLRPLIHGPALGEASPNYAANM----VI   429
AoryXlnR(1-971)      WVAAQTSDAAFLTSPPSARGRVCQKLLELTVGLLRPLIHGPAPGETSPNYAANM----VI   515
AnigXlnR(1-875)      WVAAQTSEAAFLTSPPSARGRVCQKLLELTIGLLRPLVHGPATGEASPNYAANM----VI   421
AfumXlnR             WVAAQTSDAPFLTSPPSARGRVCQKLLELTIGLLRPLIHGPAPGETSPNYAANM----VI   500
                     **.*  . *  *   *  * .:** *  **: **:**:*       .
```

```
                                                             AraR-399
AnidAraR(1-825)      NNPAFAGVGQDLPPTTVHHPFEGGGDDRGLVGPAGSLDDVITYIHVASIISSSEQKAASM   404
AoryAraR(1-830)      ---LSEPVGQELPPTTVHHPFEGGGDDRGLVGPAGSLDDVITYIHVASIISSSEQKAASM   407
AfumAraR(1-838)      SDPTFTGVAPECPPTTVHHPFESSGDDRGLVGPAGSLDDVITYIHVASIISSSEQKAASM   415
AnigAraR(1-832)      SDPLHAAVGQDGPPTTVHHPFEVGGDDRGLVGPAGSLDDVITYIHVASIISSSEQKAASM   409
TreXyr1(1-940)       GAAALGVLGVAMPG---SLNMDSLAGETGAFGAIGSLDDVITYVHLATVVSASEYKGASL   525
AnidXlnR(1-875)      NGVALGGFGVSMD---------QLGAQ---STATGAVDDVATYVHLATVVSASEYKAASM   477
AoryXlnR(1-971)      NGVALGGFGVSMD---------QLGAQ---SSATGAVDDVATYVHLATVISASEYKAASM   563
AnigXlnR(1-875)      NGVALGGFGVSMD---------QLGAQ---SSATGAVDDVATYVHLATVVSASEYKAASM   469
AfumXlnR             NGVALGGFGVSMD---------QLGAQ---SSATGAVDDVATYVHLATVISASEYKAASM   548
                            ..               . :       *::*** **:*:*:::*:** *.**:
                       AraR-416
AnidAraR(1-825)      RWWHAAFTLARELKLNQEIEVMPNGDSQVEGSSPPFGYSLPGWDGADPGPVFNYS-NPTR   463
AoryAraR(1-830)      RWWHAAFTLARELKLNQEIEVLPNVDTQTEGSSPSFDYALPGWNGVETRPFFDFS-NPTR   466
AfumAraR(1-838)      RWWHAAFTLARELKLNQEIEVIPNADGQTEGSSPAFDYSLPGWSGVDTGAFFDYS-NPTR   474
AnigAraR(1-832)      RWWHAAFTLARELKLNQEIEVMPSEENHPEGSSPSFDYSLAGWSGVDTGPFFDYS-NPAR   468
TreXyr1(1-940)       RWWGAAWSLARELKLGRELPPGNPPANQEDGEGLSED--------------VDEH-----   566
AnidXlnR(1-875)      RWWTAAWSLARELKLGRELPPNASQPGQDGERENEGD--------------NPSKRN---   520
AoryXlnR(1-971)      RWWTAAWSLARELKLGRELPPNAPQPRQDGEPEDDTD--------------VDMSKRNLP   609
AnigXlnR(1-875)      RWWTAAWSLARELKLGRELPPNVSHARQDGERDGDGE--------------A--DKRHPP   513
AfumXlnR             RWWTAAWSLARELKLGRELPPNTPHARPDAERDGDPD--------------ADLSKRHPP   594
                     *** **::*******.:*:
                                                 AraR-504
AnidAraR(1-825)      SSLNCVCDRQD--QNTITEEHREERRRTWWLLYIMDRHLALCYNRPLALLDAESEDLLLP   521
AoryAraR(1-830)      PSLNCVCDR-HDMHNTITEEHREERRRAWWLLYIMDRHLALCYNRPLALLDAESEDLLLP   525
AfumAraR(1-838)      PSLNCVCDRSHDPHATITEEHREERRRTWWLLYIMDRHLALCYNRPLALLDAESEDLLLP   534
AnigAraR(1-832)      PSLNCVCDRGHELRGAITEEHREERRRTWWLLYIMDRHLALCYNRPLALLDAESEDLLLP   528
TreXyr1(1-940)       -------DLNRNNTRFVTEEEREERRRAWWLVYIVDRHLALCYNRPLFLLDSECSDLYHP   619
AnidXlnR(1-875)      -----QSLHGGNSNVNVTEEEREERRRLWWLLYATDRHLALCYNRPLTLLDKECSQLLQP   575
AoryXlnR(1-971)      PLITSVGGNSGSTILNVTEEEREERRRLWWLLYATDRHLALCYNRPLTLLDKECEGLLQP   669
AnigXlnR(1-875)      TLITSLGHGSGSSGINVTEEEREERRRLWWLLYATDRHLALCYNRPLTLLDKECGGLLQP   573
AfumXlnR             PLITSMGHGPGNTIINITEEEREERRRLWWLLYATDRHLALCYNRPLTLLDKECEGLLQP   654
                                     :***.****** ***:*  ************ *** *.  *  *
```

```
AnidAraR(1-825)      LDEASWQSGIIHSNSPK-----SDGPQCLLSADKNKRRLFPNFICHDHSVFGFFLPLMTI   576
AoryAraR(1-830)      LDEGSWQSGNIHSNSPR-----PDGPQCLLSGDKNKRRVFPNFVCHDHSIFGFFLPLMTI   580
AfumAraR(1-838)      LDEGSWQAGNVHSNSPK-----PDGPHCPISGEKNKRRVFPDFICHDHSIFGFFLPLMTI   589
AnigAraR(1-832)      LDEGSWQSGNIHSNSPK-----PDGPQCPLSGEKNKRRVFPNFICHDHSIFGFFLPLMTI   583
TreXyr1(1-940)       MDDIKWQAGKFRSHDAGNSSINIDSSMTDEFGDSPRAARGAHYECRGRSIFGYFLSLMTI   679
AnidXlnR(1-875)      MNDDLWQAGDFPAAT---------------------YRAVGPPIECTGHSMFGYFLPLMTI   615
AoryXlnR(1-971)      MNDDLWQAGDFAGAT---------------------YRQVGPQVECTGHSMFGFFLPLMTI   709
AnigXlnR(1-875)      MNDDLWQVGDFAAAA---------------------YRQVGPPVECTGHSMYGYFLPLMTI   613
AfumXlnR             MNDDLWQAGDFA--T---------------------YRQAGPPVECTGHSMFGYFLPLMTI   692
                     :::  ** * .                                 * .:*::*:** ****

                              AraR-600
AnidAraR(1-825)      TGELIDLNQARNHPMLGMRLNGKDAWNVHVSEVLRQLEIYKASLTTFAATTSDPE-----   631
AoryAraR(1-830)      TGELIDLNQSRNHPMLGVRLNGKDAWDVHVNAVLGQLEIYKASLTTFAATASDPE-----   635
AfumAraR(1-838)      TGELIDLNQARNHPMLGSRLHGKDGWDAHLSEVLRQLEIYKASLTTFAATAAVPE-----   644
AnigAraR(1-832)      TGELIDLNQARNHPMLGARLNGKDPWDAHVGEVLRQLELYKASLTTFAATASDPD-----   638
TreXyr1(1-940)       LGEIVDVHHAKSHPRFGVGFRSARDWDEQVAEITRHLDMYEESLKRFVAKHLPLSSKDKE   739
AnidXlnR(1-875)      LGGIIDLQQAREHPRYGLTFRSGPDLDQYIMAITQQLDAYGQSLKDFEARYINSLALAEN   675
AoryXlnR(1-971)      LGEIVDLQQAKEHPRFGRVFRNSADWDHQVLEITRQLDTYAQSLKEFEARYTSSLALGAG   769
AnigXlnR(1-875)      LGGIVDLHHAENHPRFGLAFRNSPEWERQVLDVTRQLDTYGRSLKEFEARYTSNLTLGAT   673
AfumXlnR             LGEIVDLQQARNHPRFGLAFRNSAECEAQVLEIARQLDVYAQSLKEFETRYTSSLALGAA   752
                      * ::*::::..**  *  :..    :  :  :  :*: *  **. * :

                                                      AraR-671
AnidAraR(1-825)      ---APLSA-YAHAQSE---HLPAEPSLSQAYAWHTQTVISYASYLVHVLHILLVGKWDPV   684
AoryAraR(1-830)      ---APLSYAYPPPKSD---TNGVDPALTQAYSWHTQTVISYASYLVHVLHILLVGKWDPV   689
AfumAraR(1-838)      ---APLATTYRPPG-P---DPPVEPSLSQAFSWHTQTVIAYASYLVHVLHILLVGKWDPV   697
AnigAraR(1-832)      ---APLSSAFPPKP-D---QQPVEPSLAQAYSWHTQTVISYASYLVHVLHILLVGKWDPV   691
TreXyr1(1-940)       QHEMHDSGAVTDMQSPLS-VRTNASSRMTESEIQASIVVAYSTHVMHVLHILLADKWDPI   798
AnidXlnR(1-875)      E---PPENPHIDHLSPSGRSSSTVGSRVNESIVHTKMVVAYGTHIMHVLYVLLAGKWDPI   732
AoryXlnR(1-971)      ESEAAIEGSHLDHVSPSGRSTSTAGSRVNESIVHTKMVVAYGTHIMHVLHVLLAGKWDPI   829
AnigXlnR(1-875)      DNEPVVEGAHLDHTSPSGRSSSTVGSRVSESIVHTRMVVAYGTHIMHVLHILLAGKWDPV   733
AfumXlnR             ETEAAMDGSHPNHVSPSGRSSSTVESRVNESIVHTKMVVAYGTHIMHVLHILLAGKWDPI   812
                                          :       ::  *::*.::::***::**..****:
```

```
                                 XlnR-V756     XlnR-A759
AnidAraR(1-825)      SLIEDKDFWTSSPAFASTISHALDAADSVDQILRYDPDISFMPYFFGIQLLQGSFLLLLI   744
AoryAraR(1-830)      SLIEDKDFWTSSPAFASTISHALDAADSVDQILRFDPDISFMPYFFGIQLLQGSFLLLLI   749
AfumAraR(1-838)      SLIEDKDFWTSSPAFATTISHALDAADSVHQILRYDPDISFMPYFFGIQLLQGSFLLLLI   757
AnigAraR(1-832)      SLIEDKDFWTSSPAFASTISHALDAADSVDHILRYDPDISFMPYFFGIQLLQGSFLLLLI   751
TreXyr1(1-940)       NLLDDDDLWISSEGFVTATSHAVSAAEAISQILEFDPGLEFMPFFYGVYLLQGSFLLLLI   858
AnidXlnR(1-875)      NLLEDHDMWISSESFLAAMSHAVGAAEAAADILEYDPDLSFMPFFFGIYLLQGSFLLLLA   792
AoryXlnR(1-971)      NLLEDHDLWISSESFIAAMSHAVGAADAAADILEYDPDITFMPFFFGIYLLQGSFLLLLA   889
AnigXlnR(1-875)      NLLEDHDLWISSESFVSAMSHAVGAAEAAAEILEYDPDLSFMPFFFGIYLLQGSFLLLLA   793
AfumXlnR             NLLDDNDLWISSESFVAAMGHAVGAAEAAAEILEYDPDLSFMPFFFGIYLLQGSFLLLLT   872
                     .*::*.*:* ** .* :: .**:.**::  .**.:**.: ***:*:*: **********
                             AraR-763                          AraR-806  AraR-809
AnidAraR(1-825)      VERLQKEAGEGILNACEVMIRATESCVVTLNTEYQRNFRQVMRSAVAQARGR-PVNHSEI   803
AoryAraR(1-830)      VERLQKEAGEGILNACEVMIRATESCVVTLNTEYQRNFRQVMRSAVAQARGR-PVNHSEI   808
AfumAraR(1-838)      VERLQKEAGEGILNACEVMIRATESCVVTLNTEYQRNFRQVMRSAVAQARGR-PVNHSEI   816
AnigAraR(1-832)      VERLQKEAGEGILNACEVMIRATESCVVTLNTEYQRNFRQVMRSAVAQARGR-PVNHSEI   810
TreXyr1(1-940)       ADKLQAEASPSVIKACETIVRAHEACVVTLSTEYQRNFSKVMRSALALIRGRVPEDLAEQ   918
AnidXlnR(1-875)      ADKLQGDANPSVVRACETIVRAHEACVVTLNTEYQRTFRKVMRSALAQVRGRVPDDFGEQ   852
AoryXlnR(1-971)      ADKLQGDVSPSVVRACETIVRAHEACVVTLNTEYQRTFRKVMRSALAQVRGRMPEDFGEQ   949
AnigXlnR(1-875)      ADKLQGDASPSVVRACETIVRAHEACVVTLNTEYQRTFRKVMRSALAQVRGRIPEDFGEQ   853
AfumXlnR             ADKLQGDASPSVVRACETIVRAHEACVVTLNTEYQRTFRKVMRSALAQVRGRLPEDFGEQ   932
                     .::** :.. .::.***.::** *:*****.*****.* :*****:*  *** * : .*

AnidAraR(1-825)      RHRRKAVLALYRWTRKGTGLAL-  825
AoryAraR(1-830)      RHRRKAVLALYRWTRKGTGLAL-  830
AfumAraR(1-838)      RHRRKAVLALYRWTRKGTGLAL-  838
AnigAraR(1-832)      RHRRKAVLALYRWTRKGTGLAL-  832
TreXyr1(1-940)       QQRRRELLALYRWTGNGTGLAL-  940
AnidXlnR(1-875)      QQRRREVLSLYRWTGDGTGLALS  875
AoryXlnR(1-971)      QQRRREVLALYRWTGDGSGLAL-  971
AnigXlnR(1-875)      QQRRREVLALYRWSGDGSGLAL-  875
AfumXlnR             QQRRREVLALYRWTGDGSGLAL-  954
                     ::**: :*:****: .*:****
```

Notes: The Zn2Cys6 domain DNA binding domain is indicated in green, In yellow the amino acids, their position in the *A. niger* AraR or XlnR proteins, and their conservation in AraR and XlnR in other filamentous fungi is indicated.
